# Local Enhancement Promotes Cockroach Feeding Aggregations

**DOI:** 10.1371/journal.pone.0022048

**Published:** 2011-07-19

**Authors:** Mathieu Lihoreau, Colette Rivault

**Affiliations:** Unité Mixte de Recherche 6552, Centre National de la Recherche Scientifique, Université de Rennes 1, Rennes, France; AgroParisTech, France

## Abstract

Communication and learning from each other are part of the success of animal societies. Social insects invest considerable effort into signalling to their nestmates the locations of the most profitable resources in their environment. Growing evidence also indicates that insects glean such information through cues inadvertently provided by their conspecifics. Here, we investigate social information use in the foraging decisions by gregarious cockroaches (*Blattella germanica* L.). Individual cockroaches given a simultaneous choice in a Y-olfactometer between the odour of feeding conspecifics and the mixed odour of food plus non-feeding conspecifics showed a preference for the arm scented with the odour of feeding conspecifics. Social information (the presence of feeding conspecifics) was produced by cockroaches of all age classes and perceived at short distance in the olfactometer arms, suggesting the use of inadvertently provided cues rather than signals. We discuss the nature of these cues and the role of local enhancement (the selection of a location based on cues associated with the presence of conspecifics) in the formation of feeding aggregations in *B. germanica*. Similar cue-mediated recruitments could underpin a wide range of collective behaviours in group-living insects.

## Introduction

Group-living provides animals with the opportunity to learn from each other. Social information is transmitted in the form of ‘signals’ shaped by natural selection to convey particular messages, or ‘cues’ inadvertently provided as a coincidental by-product of others' behaviour or metabolic activity [1], [2]. Both types of information transfer can give rise to social learning, from simple local enhancement when an individual is attracted to a particular object or a particular environmental zone [3], to more complex transmissions of cultural skills or knowledge [4].

Although the study of social information use has long been limited to vertebrates, communication and learning from others are widely recognized as part of the success of insect societies [5], [6]. For instance, social insects invest considerable effort into passing on learnt information about the location and/or the quality of available resources to their nestmates in the form of signals. This is epitomized by complex recruitment behaviours underpinning the collective selection of feeding or resting sites in highly integrated colonies of honeybees [7], [8], ants [9], [10] and caterpillars [11]. Growing evidence also indicate that insects glean social information using cues inadvertently provided by their conspecifics to avoid predation risks [12], choose mating partners [13] or assess food quality [14]–[16]. Contrary to active signalling, information transfer through social cues should occur in a wide range of contexts, from situations where unintentional signallers benefit substantially by doing so, to situations where they gain no benefit or even incur some costs [17]. Therefore, we would expect inadvertent social information use to be widespread in group-living insects and play a key role in shaping social interactions in a wide range of species, including species exhibiting low levels of social cohesion [18].

We explored this possibility in the gregarious cockroach *Blattella germanica* (L.). German cockroaches live in mixed-family groups (0<*r*≤0.5) where individuals of all developmental stages (sex-ratio 1∶1) share a common resting shelter [19]. Nymphs and adults are omnivorous generalists and usually feed on the same food sources [20]. *B. germanica* is a central place forager species. Individuals exploit food sources on site and return to their resting shelter after each foraging event. Under low population densities, cockroaches forage independently from each others, search for and exploit food sources based on the knowledge of their home range and detection of food odours [21], [22]. However, under large population densities, cockroaches form feeding aggregations, suggesting that they use social information in their individual foraging decisions [23]. Mathematical models used to describe these collective foraging dynamics indicate that recruitment of foragers to a food source is more likely to be mediated by short-range than by long-range information transfer, so that that the presence of conspecifics on food patch does not influence the probability of a forager to join this particular patch (no conspecific attraction), but increases its probability to stay when the patch has been discovered (conspecific retention) [23]. However, there is no clear empirical evidence for this.

In this study, we investigated the use of social information in foraging decisions by individual cockroaches. We performed odour choice experiments in a Y-olfactometer to investigate (a) whether cockroaches use social information to select food sources, (b) whether this information is produced and perceived by cockroaches of all age classes and both sexes, and (c) whether this information remains available on food sources for long durations (after the removal of conspecifics).

## Materials and Methods

Experimental subjects were *B. germanica* cockroaches from our laboratory stock culture (UMR 6552, Rennes, France). Mature oothecae were collected from gravid females and placed in individual plastic rearing boxes (diameter = 80 mm; height = 50 mm), where they hatched within 24–48 h. Siblings from each ootheca (approximately 35 1^st^ instar nymphs; sex-ratio 1∶1) were maintained in these boxes until being tested, thus providing social interactions necessary to stimulate their development [24], [25]. Cockroaches were reared under controlled illumination (12 h light-12 h dark cycle) and stable temperature (25°C). They were provided with *ad libitum* turkey food pellets, water and cardboard shelters.

### Olfactometer

**Figure 1:**
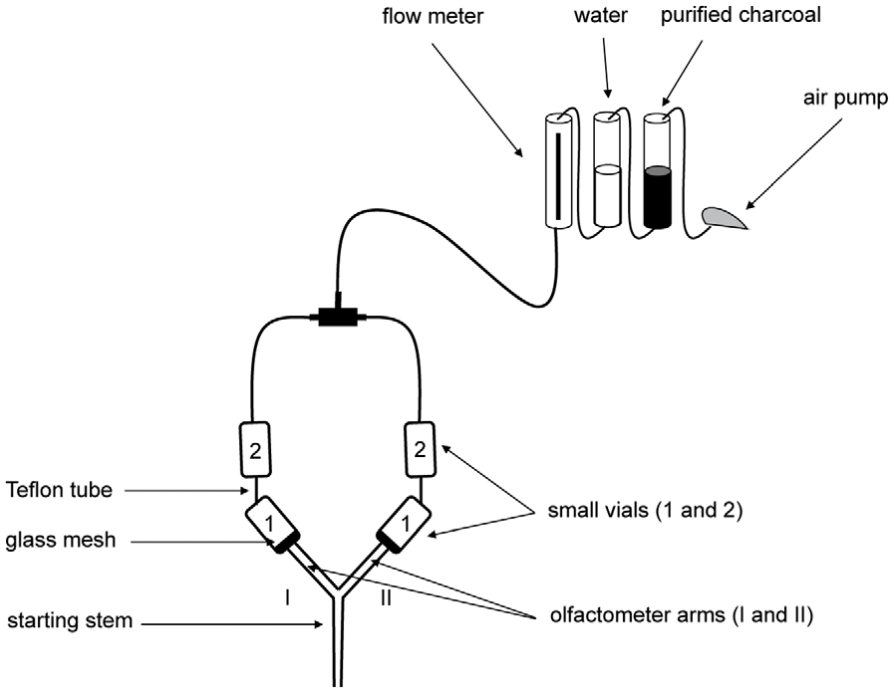
Olfactometer. Individual cockroaches were given a simultaneous choice between two stimulus odours in a glass Y-olfactometer. A pump pushed clean controlled humidified air through the four small vials, the two arms and the starting stem of the olfactometer. Each arm (arms I, II) was connected to two vials placed in a row (vials 1, 2). Vials were either empty (A: clean air), or contained fresh food (F), recently consumed food (NF), non-feeding conspecifics (C), or feeding conspecifics (FC). Choice of the test cockroach for a stimulus odour (odour 1 or 2) was assessed by the total time spent in each olfactometer arm during 5 min tests (see details in Table 1).

We used a glass Y-olfactometer (Fig. 1) composed of a starting stem (internal diameter = 10 mm; length = 100 mm) and two lateral arms (60° apart; internal diameter = 10 mm; length = 100 mm). Each arm (arms I, II) was connected to two small glass vials (internal diameter = 20 mm; length = 80 mm) placed in a row (vials 1, 2). A pump (New-air, Lorregia, Italy) connected to a flow meter (Brooks, Hatfield, PA) pushed charcoal-purified humidified air at a constant flow rate (60 ml.min^−1^) through the four vials and the two olfactometer arms. Connections between the arms and the vials were made with a fritted glass filter (mesh diameter = 1 µm; thickness = 1 mm), thus homogenizing odours flow from the vials throughout the arms, whilst preventing test cockroaches from having access to the vials. Vials were covered with black clothes and connected to each other with Teflon tubes (internal diameter = 0.5 mm; length = 100 mm long). This setup strictly precluded test cockroaches to detect the contents of the vials through vision or physical contacts. Although the use of sonic communication was possible, conduction of vibrational information from the vials to the olfactometer might have been highly disturbed by multiple physical barriers (i.e. Teflon tube sections, fritted glass filter).

### Choice experiments

#### Subjects

Odour choice experiments were performed with young nymphs (2^nd^ instar nymphs, 10 days old), old nymphs (6^th^ instar nymphs, 45 days old), adult females (70 days old), and adult males (70 days old). All subjects were deprived of food during the five days preceding the tests. They were used either as ‘test individuals’ (individuals given a choice in the olfactometer), or ‘stimulus individuals’ (enclosed in glass vials for the production of stimulus odours).

#### Stimulus odours

Test individuals were given a simultaneous choice between two stimulus odours in the olfactometer. Each stimulus odour emanated from food and/or conspecifics enclosed in the two vials (1, 2) connected to an olfactometer arm (see Table 1). Vials were either empty (A: clean air), or contained fresh food (F: 280 mg of fresh food pellets deposited just before tests), recently consumed food (NF: 280 mg food pellets on which 15 cockroaches fed for 30 minutes immediately before the test), non-feeding conspecifics (C: 15 cockroaches), or feeding conspecifics (FC: 15 cockroaches feeding on 280 mg fresh food pellets). A mixed odour of food plus non-feeding conspecifics (F+C) was obtained using food and conspecifics in the two separate vials. Because *B. germanica* cockroaches preferentially interact with siblings rather than with non-siblings in a social context [26], all cockroaches were tested with odours of non-siblings (i.e. conspecifics issued from a different ootheca than that of test individuals). This procedure precluded genetic relatedness to act as a confounding variable in the observed choices. Preliminary experiments indicated that cockroaches discriminate and prefer the odours of F, C and FC, to a clean air flow A, thus validating the choice of our stimulus odours (Table S1).

**Table 1:** Design of choice experiments.

Exp	Replicates	*n*	Test individuals	Conspecifics used as stimulus	Stimulus odour 1	Stimulus odour 2
1	74	72	old nymph	young nymphs	F+C	FC
2	56	55	old nymph	old nymphs	F+C	FC
3	54	53	old nymph	adult female	F+C	FC
4	74	74	old nymph	adult male	F+C	FC
5	64	64	young nymph	old nymphs	F+C	FC
6	62	61	adult female	old nymphs	F+C	FC
7	94	93	adult male	old nymphs	F+C	FC
8	100	97	old nymph	old nymphs	F	NF

In each experiment, a test individual was given a simultaneous choice between two stimulus odours in a Y-olfactometer (Fig. 1). Each stimulus odour emanated from food and/or conspecifics enclosed in the two vials (1, 2) connected to the olfactometer arm. F: fresh food in vial 1+empty vial 2; NF: recently consumed food in vial 1+empty vial 2; F+C: non-feeding conspecifics in vial 1+fresh food in vial 2; FC = feeding conspecifics in vial 1+empty vial 2. Replicates: total number of tests. *n*: number of successful tests (tests during which the cockroach visited both olfactometer arms).

#### Procedure

We designed eight choice experiments (see details in Table 1) to investigate (a) whether foraging cockroaches use social information to select food sources (exp. 1–4), (b) whether this information is produced and perceived by individuals of all age classes and both sexes (exp. 5–7), and (c) whether this information remains available on food sources for long durations (after the removal of conspecifics; exp. 8). Based on our previous results suggesting that 6^th^ instar nymphs select food sources in relation to the number of conspecifics already feeding on it [23], we used old nymphs as a control either as test individuals or as stimulus individuals to investigate cockroach ability to produce and/or perceive social information at different age classes (young nymphs, adult males or adult females). Each cockroach was tested only once.

- In experiments 1–4, old nymphs were given a choice between FC and F+C. Stimulus individuals were either young nymphs (exp. 1, 74 replicates), old nymphs (exp. 2, 56 replicates), adult females (exp. 3, 54 replicates) or adult males (exp. 4, 74 replicates).
- In experiments 5–7, young nymphs (exp. 5, 64 replicates), adult females (exp. 6, 62 replicates) or adult males (exp. 7, 94 replicates) were given a choice between FC and F+C. Stimulus individuals were old nymphs.
- In experiment 8, old nymphs were given a choice between F and NF. Stimulus individuals (individuals that fed on the food source previous to the test) were old nymphs (100 replicates).

The day prior to the observations, test cockroaches were isolated in small plastic tubes (internal diameter = 10 mm; length = 30 mm). Groups of stimulus cockroaches (15 siblings) were placed in the olfactometer vials just before the tests, in order to avoid chemical marking [27]. Observations were performed under red light during the night phase of the photocycle. Test cockroaches were released from their plastic tubes directly at the entrance of the olfactometer starting stem to avoid stress due to manipulation by the experimenter. From then on, we recorded the time each individual visited the arms and the duration of each visit for 5 minutes. When feeding conspecifics were used as stimulus individuals, we recorded the number of cockroaches that were feeding at the beginning and at the end of the test. This allowed us to estimate the amount of social information available for the test individual during the observations. A test was successful only when the cockroach explored both olfactometer arms [28]. After the observations, cockroaches were removed from the olfactometer and both arms were washed (99% dichloromethane) to remove chemical scents. To avoid potential biases due to an inherent tendency by cockroaches to turn right or left in the olfactometer, or to irregularities in the air flow between the two branches, for each experiment we performed half of the tests with stimulus odour 1 in arm I and stimulus odour 2 in arm II. Conversely, the other half of the tests was performed with stimulus odour 2 in arm I and stimulus odour 1 in arm II (Fig. 1; Table 1).

### Data analysis

Data were analysed using R 2.10.1. [29]. We excluded unsuccessful tests (replicates when the cockroaches did not explore the two olfactometer arms) from the dataset. These tests were rare and equally distributed among experiments (mean = 1.50±0.39 (s.e.) test per experiment; *χ* ^2^_7_ = 3.57, *p* = 0.828; Table 1). To control for family-wise error rates associated with multiple comparisons, we performed single analyses combining the data from experiments 1–7 and a separate analysis on the data of experiment 8. This way, we compared the frequencies of cockroaches' first choice (first arm visited) to each arm using binomial tests (random probability 0.5), the average number of visits to each arm using Wilcoxon tests, the total time spent in each arm and the average duration of each visit using *t*-tests. We then made comparisons between experiments using Generalized Linear Models (GLM). We examined the effects of experimental treatment (experiments 1–8), the effect of the quality of social information (type of stimulus individuals: young nymphs, old nymphs, adult females, adult males), the effect of the amount of available social information (number of feeding stimulus individuals during the observations), and the effect developmental stage/gender of test individual used (type of test individuals: young nymphs, old nymphs, adult females, adult males), on the total time spent in the FC scented arm. For each model, we selected the error structure family in relation to the nature of the dependent variable (binomial error structure for proportion data, Poisson error structure for non-normally distributed count data, Gaussian error structure for normally distributed data). Non-combined data (data for each individual experiment) are showed in Table 2. All means are given with standard error.

**Table 2:** Results of choice experiments (non-combined data).

		Total time spent in arm (mean ± s.e., in s)		Number of first visits		Average number of visits (mean ± s.e.)		Average visit duration (mean ± s.e., in s)	
Exp	*n*	Stimulus odour 1	Stimulus odour 2	Stimulus odour 1	Stimulus odour 2	Stimulus odour 1	Stimulus odour 2	Stimulus odour 1	Stimulus odour 2
1	72	62.00±3.72	77.63±5.65	42	30	3.60±0.17	3.60±0.16	22.40±2.98	28.00±4.27
2	55	59.10±3.50	83.76±5.58	27	28	3.44±0.18	3.69±0.20	21.92±2.47	27.92±3.99
3	53	58.25±3.65	82.29±4.91	34	19	4.42±0.30	4.91±0.34	18.00±1.51	20.33±1.29
4	74	59.34±3.75	72.42±4.59	36	38	3.26±0.15	3.53±0.15	20.24±1.37	23.90±1.98
5	64	70.70±4.68	85.40±4.64	33	31	4.05±0.22	4.25±0.24	21.67±1.34	26.50±2.50
6	61	53.33±3.20	75.08±4.32	30	31	3.39±0.18	3.80±0.19	14.05±0.59	22.00±1.32
7	93	63.39±3.30	73.05±3.35	54	39	4.62±0.24	4.38±0.16	17.55±0.86	22.55±1.47
8	97	70.29±2.89	76.34±4.33	47	50	4.76±0.18	4.62±0.17	16.59±0.77	25.61±3.34

**Figure 2:**
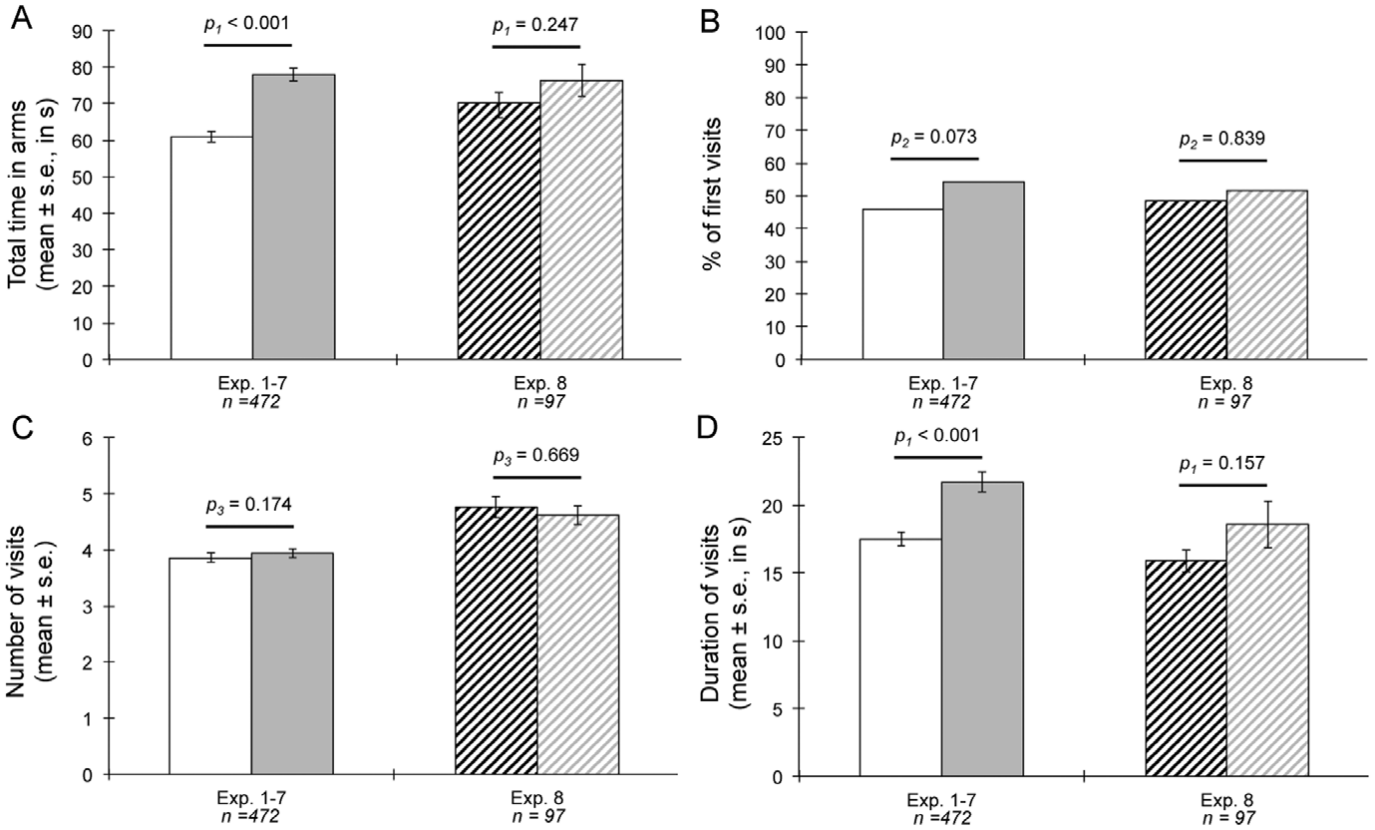
Results of choice experiments (combined data). A. total time spent in each olfactometer arm; B. percentage of first visits to each arm; C. average number of visits to each arm; D. average duration of visits to each arm. White bar: arm scented with food plus non-feeding conspecifics (F+C); grey bar: arm scented with feeding conspecifics (FC); black-striped bar: arm scented with fresh food (F); grey-striped bar: arm scented with recently consumed food (NF); *n*: number of successful tests (tests during which the cockroach visited both olfactometer arms). *p_1_*: *t*-test; *p_2_*: binomial test *p_3_*: Wilcoxon test. Non-combined data (data for each individual experiment) are showed in Table 2.

For each experiment, the total time, the numbers of first visits, the average numbers of visits, and the average durations per visit of test cockroaches in each olfactometer arm are showed. Exp. 1–7: stimulus odour 1 = food plus non-feeding conspecifics (F+C), stimulus odour 2 = feeding conspecifics (FC); exp. 8: stimulus odour 1 = fresh food (F), stimulus odour 2 = recently consumed food (NF); *n*: number of successful tests (tests during which the cockroach visited both olfactometer arms). Statistical analyses performed on combined data are showed in Fig. 2.

## Results

### Odour choices

Cockroaches given a choice between FC and F+C (exp. 1–7) spent significantly more time in the FC scented arm, indicating that they used social information from feeding conspecifics in their foraging decisions (Fig. 2A). This tendency was consistent across experiments whatever the type of test individuals and stimuli individuals we used (Table 2). Comparison of data from experiments 1–4 indicate that old nymphs spent similar time visiting the FC scented arm when tested with young nymphs (exp. 1), old nymphs (exp. 2), adult males (exp. 3) or adult females (exp. 4) as stimulus individuals and whatever the number of feeding stimulus individuals (GLM, Gaussian error structure, type of stimulus individuals: *F*_3,246_ = 1.57, *p* = 0.197, number of stimulus individuals: *F*_1,246_ = 0.05, *p* = 0.831, interaction: *F*_3,246_ = 1.17, *p* = 0.323). Thus, social information was produced by individuals at all developmental stages and both genders. Test cockroaches were more sensitive to the quality (presence/absence of feeding conspecifics) than to the amount (number of feeding conspecifics) of available information. Comparison of data from experiments 2, 5–7 indicate that young nymphs (exp. 5), old nymphs (exp. 2), adult males (exp. 6) and adult females (exp. 7) spent similar time in the FC scented arm when tested with old nymphs as stimulus individuals (GLM, Gaussian error structure, type of stimulus individuals: *F*_3,269_ = 1.06, *p* = 0.369). Therefore, social information was perceived by individuals at all developmental stages and both genders. The fact that cockroaches given a choice between F and NF (exp. 8) did not discriminate between the two options indicates that social information was no longer available after the removal of conspecifics from the food source (Fig. 2A, Table 2).

### Dynamics of choices

Detailed analyses of visits to each olfactometer arm indicate that cockroaches given a choice between FC and F+C (exp. 1–7) first explored both arms randomly (Fig. 2B, Table 2). The lack of significant first choice reveals that social information was not perceived at a long distance in the starting stem of the olfactometer but only at very short distance from the stimulus odour source in the olfactometer arms. On average, test cockroaches made similar numbers of visits to both arms (Fig. 2C, Table 2). However, visits were longer in the FC scented arm than in the F+C scented arm (Fig. 2D, Table 2). Thus, observed choices arose from the accumulation of longer visits in the FC scented arm than in the F+C scented arm. We found no significant difference between experiments in the percentage of first visits to the FC arm (GLM, binomial error structure, proportion of first visit to FC: *p* = 0.4814), the number of visits to the FC arm (GLM, Poisson error structure, average number of visits to FC: *p* = 0.1372), and the average duration of visits to the FC arm (GLM, Gaussian error structure, average visit duration to FC: *F*_6,465_ = 1.79, *p* = 0.100). Analyses of visits to each arm in experiment 8 confirm the absence of a significant discrimination between F and NF (Figs. 2B–2D, Table 2). Cockroaches made random first choices, similar numbers of visits and spent similar time in each olfactometer arm.

## Discussion

Our study demonstrates that *B. germanica* cockroaches use social information when making foraging decisions, most possibly through low volatile cues inadvertently provided by feeding conspecifics rather than signals. We discuss the nature of these social cues and the role of local enhancement as a mechanism promoting cockroach feeding aggregations.

Growing evidence highlights that insects, like vertebrates, commonly use social cues to update their knowledge of the environment [6]. This is perhaps best demonstrated in pollinating bees and wasps, where naive foragers copy more experienced conspecifics to learn the location and the phenotype of the most rewarding flowers [14]–[16], [30]. Our findings not only add a new species to the list of insects using inadvertent social information, but suggest that local enhancement (the attraction of an individual to a location through cues associated with the presence of conspecifics) can underpin complex group dynamics such as collective selection of food sources by cockroaches [23]. In our experimental setup, hungry cockroaches preferred joining feeding than non-feeding conspecifics. The fact that their first visit to an olfactometer arm was random suggests that social information associated to the presence of feeding conspecifics was not perceived over long-distance (in the starting stem of the olfactometer) but rather at short distance (a few centimetres) from the stimulus group. Short-range perception of low-volatile cues involved in the selection of resting partners by cockroaches has previously been identified using similar olfactometer devices [26], [31]. If feeding cockroaches used signals shaped by natural selection to advertise the location and/or the quality of food sources for their conspecifics, we would expect the information to be perceived at longer distance, thus increasing the probability of foraging individuals to join [17]. Instead, cockroaches seem to assess the presence of feeding conspecifics through inadvertently provided cues only once arrived in the vicinity of the food source. This observation is in accordance with predictions of our mathematical model, suggesting that feeding aggregations in *B. germanica* arise from short-range information transfer through a retention effect of feeding individuals on newcomers at the food source [23]. Therefore, there is little doubt that the local enhancement process we describe at the individual level (the selection of a food source based on cues associated with the presence of feeding conspecifics) acts as a grouping mechanism underpinning the formation of feeding aggregations and the occurrence of collective food choices observed in this species.

Although, this first exploration of social information use by foraging cockroaches was not designed to identify the cues involved in local enhancement, our results allow us to restrict the list of potential candidates. Our setup strictly precluded the use of visual and tactile communication as the vials containing feeding conspecifics were covered with black clothes and physically separated from the olfactometer arms. In addition, although *B. germanica* cockroaches are sensitive to vibrations and can use auditory cues to detect resting aggregates [32], [33], sonic communication was unlikely to occur through the setup as the multiple physical barriers between the vials and the olfactometer arms might have highly disturbed any vibrational transmission. Therefore, our results suggest that cockroaches based their foraging decisions on olfactory cues emanating from feeding conspecifics.

According to predictions of foraging theory, group foraging may rapidly become a suboptimal strategy when population density increases, as any departure from an ideal free distribution on available sources should strengthen competition [34], [35]. Thus, what are the benefits of joining feeding conspecifics? First of all, grouping is a taxonomically widespread anti-predation strategy. In the case of cockroaches, both dilution and confusion effects might increase individual's probability of escaping safely under human threat [36]. Second, social cohesion during resting phases is essential for cockroaches to maintain body warmth [24] and reduce water loss through evaporation [37]. Group foraging could be also a strategy to extend these physiological benefits to the activity phases. Third, the fact that groups are composed of individuals at different developmental stages might benefit to young nymphs as adults and old nymphs have the potential to process foods and break mechanical barriers that prevent small individuals from feeding on certain types of food [38]. But perhaps more importantly, local enhancement might allow cockroaches to share essential information about available food sources. In urban environments, *B. germanica* cockroaches forage on highly unpredictable and patchily distributed resources [19]. The presence of conspecifics on a food source is therefore an honest message that guarantees its quality and/or quantity and foragers may increase their detection speed of food location and their accuracy in food quality assessment by simply joining the group. Optimization of speed and accuracy of choices is a key prediction of collective-decisions making processes [39]. In the case of omnivorous cockroaches, which have to mix their diet from foods that vary greatly in composition [40], social information transfer might help individuals to select best profitable foods, balance their nutritional intake, and eventually avoid toxic components. How all these ecological benefits of grouping interplay and favour the formation of cockroach feeding aggregations remains however unclear and needs to be tested.

Identifying whether information is actively shared or inadvertently provided by individuals brings fundamental insights into the understanding of the mechanisms and the evolution of animal behaviours. Our study suggests that the use of social cues through local enhancement leads to the formation of feeding aggregation and mediates collective foraging decisions in cockroaches. Further exploration of the role of inadvertent social information use in shaping social interactions in insects groups might help improving our understanding of the evolution of group coordination and social behaviours.

## Supporting Information

Table S1 **Relevance of stimulus odours.** In each experiment, a test individual was given a simultaneous choice between a clean air flow and a stimulus odour in a Y-olfactometer (Fig. 1). The stimulus odour emanated from food and/or conspecifics enclosed in the two vials (1, 2) connected to one of the two olfactometer arms. F: fresh food in vial 1+empty vial 2; C: non-feeding conspecifics in vial 1+empty vial 2; FC: feeding conspecifics in vial 1+empty vial 2. In all three experiments, test cockroaches spent significantly more time in the arm scented with the stimulus odour than in the arm containing clean air, indicating that all stimuli were attractive and relevant to investigate cockroach foraging behaviour. *p*: *t*-test.
(DOC)
