# Supplementary material for: Local Enhancement Promotes Cockroach Feeding Aggregations
Source: PLoS One. 2011 Jul 19;6(7):e22048. doi: 10.1371/journal.pone.0022048 (PMC3139617; doi:10.1371/journal.pone.0022048)
Supplement: Table S1 — Relevance of stimulus odours. In each experiment, a test individual was given a simultaneous choice between a clean air flow and a stimulus odour in a Y-olfactometer (Fig. 1). The stimulus odour emanated from food and/or conspecifics enclosed in the two vials (1, 2) connected to one of the two olfactometer arms. F: fresh food in vial 1+empty vial 2; C: non-feeding conspecifics in vial 1+empty vial 2; FC: feeding conspecifics in vial 1+empty vial 2. In all three experiments, test cockroaches spent significantly more time in the arm scented with the stimulus odour than in the arm containing clean air, indicating that all stimuli were attractive and relevant to investigate cockroach foraging behaviour. p: t-test. (DOC) [file pone.0022048.s001.doc]

**Table S1.** **Relevance of stimulus odours.**

| Exp | *n* | Stimulus odour | Total time spent in arm containing clean air (mean ± s.e., in s) | Total time spent in arm scented with the stimulus odour (mean ± s.e., in s) | *p* |
| --- | --- | --- | --- | --- | --- |
| 1 | 30 | F | 81.83 ± 8.67 | 131.97 ± 8.60 | < 0.001 |
| 2 | 30 | C | 74.90 ± 8.09 | 138.80 ± 7.51 | < 0.001 |
| 3 | 30 | FC | 85.47 ± 8.50 | 128.23 ± 9.41 | 0.001 |
